# The bisphosphonate zoledronic acid impairs membrane localisation and induces cytochrome *c* release in breast cancer cells

**DOI:** 10.1038/sj.bjc.6600297

**Published:** 2002-05-06

**Authors:** S G Senaratne, J L Mansi, K W Colston

**Affiliations:** Department of Oncology, Gastroenterology, Endocrinology and Metabolism, St George's Hospital Medical School, Cranmer Terrace, London SW17 ORE, UK

**Keywords:** bisphosphonates, breast cancer, apoptosis, caspases, Ras, cytochrome *c*

## Abstract

Bisphosphonates are well established in the management of cancer-induced bone disease. Recent studies have indicated that these compounds have direct inhibitory effects on cultured human breast cancer cells. Nitrogen-containing bisphosphonates including zoledronic acid have been shown to induce apoptosis associated with PARP cleavage and DNA fragmentation. The aim of this study was to identify the signalling pathways involved. Forced expression of the anti-apoptotic protein bcl-2 attenuated bisphosphonate-induced loss of cell viability and induction of DNA fragmentation in MDA-MB-231 cells. Zoledronic acid-mediated apoptosis was associated with a time and dose-related release of mitochondrial cytochrome *c* into the cytosol in two cell lines. Rescue of cells by preincubation with a caspase-3 selective inhibitor and demonstration of pro-caspase-3 cleavage products by immunoblotting suggests that at least one of the caspases activated in response to zoledronic acid treatment is caspase-3. In both MDA-MB-231 and MCF-7 breast cancer cells, zoledronic acid impaired membrane localisation of Ras indicating reduced prenylation of this protein. These observations demonstrate that zoledronic acid-mediated apoptosis is associated with cytochrome *c* release and consequent caspase activation. This process may be initiated by inhibition of the enzymes in the mevalonate pathway leading to impaired prenylation of key intracellular proteins including Ras.

*British Journal of Cancer* (2002) **86**, 1479–1486. DOI: 10.1038/sj/bjc/6600297 www.bjcancer.com

© 2002 Cancer Research UK

## 

Breast cancer commonly metastasises to bone: over 80% of women with advanced breast cancer develop bone metastases which ultimately account significantly for morbidity and mortality. Bisphosphonates (BPs) are potent inhibitors of bone resorption and are effective in the treatment of many metabolic bone diseases. In animal models of metastatic disease, BPs have been shown to slow the development of bone metastases and to reduce tumour burden in bone (Wingen *et al*, 1986; Yoneda *et al*, 2000). In patients with advanced breast cancer and bone metastases, BPs reduce the incidence of hypercalcaemia and skeletal morbidity (Ryzen *et al*, 1985). In addition, a recently reported clinical trial of patients with breast cancer has suggested that the BP clodronate given in the adjuvant setting can reduce the incidence of skeletal metastases with a consequent improvement in survival (Diel *et al*, 1998). Such observations could indicate that BPs have direct inhibitory effects on breast cancer cells. This suggestion is supported by the demonstration that BPs inhibit breast cancer cell adhesion to bone *in vitro* (van der Pluijm *et al*, 1996; Boissier *et al*, 1997).

We have recently demonstrated that two amino bisphosphonates (N-BPs), pamidronate and zoledronic acid (ZOL), directly inhibit growth and viability of cultured human breast cancer cells. These effects are accompanied by morphological changes, induction of DNA fragmentation, decreased bcl-2/bax ratio and PARP cleavage indicative of caspase activation (Senaratne *et al*, 2000). These findings have been confirmed by reports from two other groups that provide evidence for caspase activation in N-BP-induced apoptosis in breast cancer cells (Fromigue *et al*, 2000; Hiraga *et al*, 2001). Initiation of the caspase cascade in response to many apoptotic stimuli is a consequence of cytochrome *c* release from mitochondria into the cytosol (Li *et al*, 1997; Zou *et al*, 1997). It has been suggested that bcl-2 prevents the release of cytochrome *c*, thereby inhibiting the activation of caspases (Marzo *et al*, 1998). One of the aims of the present study was to clarify the involvement of bcl-2 and cytochrome *c* in caspase activation induced by the N-BP, ZOL in breast cancer cells. In this study we show for the first time that treatment of breast cancer cells with ZOL is associated with release of mitochondrial cytochrome *c* into the cytosol consistent with a decrease in the action of bcl-2. To further support a relationship between bcl-2 and N-BP mediated apoptosis, we now report that forced expression of bcl-2 abrogates ZOL-induced DNA fragmentation which is a consequence of caspase activation in breast cancer cells.

In order to identify further the mechanisms initiating this caspase activation leading to N-BP-induced apoptosis in breast cancer cells, we evaluated the possible role of impaired protein prenylation. Recent pharmacological studies suggest that N-BPs such as pamidronate, alendronate and risedronate act on the enzymes in the mevalonate pathway leading to decreased generation of isoprenoid intermediates required for post-translational prenylation of key cellular proteins. This is the suggested mechanism by which N-BPs mediate apoptosis in macrophages and myeloma cells (Luckman *et al*, 1998; Shipman *et al*, 1998). However, the identity of the proteins that are ineffectively prenylated following N-BP treatment of breast cancer cells is presently unknown. Ras proteins play pivotal roles in the control of normal and transformed cell growth. For functional activity, Ras requires membrane localisation and this is mediated by post-translational modification by the addition of a 15-carbon farnesyl isoprenoid. By inhibiting farnesyl pyrophosphate (FPP) generation, N-BPs might thus impair normal farnesylation of Ras. In this study we present support for this potential mechanism and report for the first time that treatment with ZOL leads to impaired membrane localisation of Ras protein in breast cancer cells that express both wild-type and mutant *ras*.

## METHODS

### Cell lines

The breast cancer cell lines MDA-MB-231 (provided by Professor RC Coombes, Imperial College, UK), and MCF-7 (Danish Cancer Society, Copenhagen, Denmark), were maintained in Dulbecco's Modified Eagle Medium (DMEM) supplemented with 100 U ml^−1^ streptomycin and 5% foetal calf serum (FCS) at a constant temperature of 37^o^C with a humidified atmosphere of 5% CO_2_. Cells were routinely tested for mycoplasma contamination. Caspase-3 expressing MCF-7 cells, a kind gift from Professor Alan Porter (The National University of Singapore, Singapore), were maintained as above together with 200 μg ml^−1^ G418 sulphate (Geneticia, Life Technologies, Paisley, UK).

### Reagents

Disodium salt of zoledronic acid ((1-hydroxy-2-imidazol-1-yl-phosphonoethyl) phosphonic acid) was obtained from Novartis Pharmaceuticals Limited (Basle, Switzerland). Cell permeable caspase-3 inhibitor 1 (Ac-Ala-Ala-Val-Ala-Leu-Leu-Pro-Ala-Val-Leu-Leu-Ala-Leu-Leu-Ala-pro-Asp-Glu-Val-Asp-CHO) and tumour necrosis factor alpha (TNF-α) were obtained from Calbiochem, CN Biosciences (Nottingham, UK). Farnesol (3,7,11-Trimethyl-2-6,10-dodecatrien-1-ol, mixed isomers), and *trans, trans*-geranylgeraniol (all *trans*-3,7,11,15-tetramethyl-2,6,10,14-hexadecatetraenyl pyrophosphate) were obtained from Sigma-Aldrich Company Ltd (Poole, Dorset, UK). The pUSEamp(+) plasmid containing wild-type mouse bcl-2 under the control of the cytomegalovirus promoter was obtained from Upstate Biotechnology (Lake Placid, NY, USA). A control vector without the insert was obtained from the same source.

### Measurement of cell viability

Cell viability was determined by 3-(4,5-dimethylthiazol-2-yl)-5-(3-carboxymethoxyphenyl)-2-(4-sulfophenyl)-2H-tetrazolium (MTS) dye reduction assay measuring mitochondrial respiratory function (Cory *et al*, 1991). Breast cancer cells (2×10^3^ per well) were plated into 96-well plates and treated with reagents or vehicle. At the end of the treatment period, MTS dye (2 mg per 20 μl per well) was added to the cell culture medium for 2 h. Absorbance was determined in a Titertek plate reader at 492 nm.

### Immunoblotting

Breast cancer cells were harvested by scraping. Whole cell lysates were prepared by washing the cells in ice cold phosphate buffered saline (PBS) and resuspending in 100 μl of lysis buffer (20 mM Tris, 40 mM sodium phosphate, 50 mM sodium fluoride, 5 mM magnesium chloride, 10 mM ethylene glycol-bis(b-aminoethyl ether) N,N,N′,N′-tetraacetic acid (EGTA), 0.5% sodium deoxycholate, 1% Triton X-100, 0.1% SDS, 40 mg ml^−1^ leupeptin, 100 mg ml^−1^ aprotinin, 20 mg ml^−1^ PMSF in Dimethyl sulphoxide (DMSO) and 50 mM sodium orthovanadate) for 30 min on ice. Equivalent protein extracts (28 μg) from each sample were electrophoresed on 16% SDS-polyacralamide gels and proteins were immobilised by transfer onto nitrocellulose membranes. Membranes were immunoprobed with 5 μl ml^−1^ equivalent of rabbit polyclonal antibodies against β actin (Sigma-Aldrich Company LTD, UK) and caspase-3 (Santa Cruz, Heidelberg, Germany) followed by a horseradish peroxidase-conjugated secondary antibody to rabbit immunoglobulins (Amersham International, UK). Specific bands were visualised by enhanced chemiluminescence (ECL, Amersham International, UK).

To identify changes in membrane localisation of Ras, breast cancer cells were treated with N-BPs or vehicle for 3 days and harvested by scraping. Cells were disrupted by homogenisation followed by five passes through a 25 gauge needle. Cytosolic and membrane fractions were separated by centrifugation at 43 000 r.p.m. for 25 min as previously described (Mumby *et al*, 1990). After solubilising in 0.01% SDS, the soluble fractions were concentrated using Micron 10 microconcentrators (Vivascience, Sartorius group, Goettingen, Germany). Equivalent protein extracts (10 μg) of cytosol and membrane fraction from each sample were electrophoresed on 12% SDS–PAGE mini gels. A mouse monoclonal primary antibody generated against Ha-Ras protein p21, which recognises wild-type and mutant Ras forms (Transduction Laboratories, CA, USA) was used to immunoprobe membranes. Proteins were detected using a horseradish peroxidase-conjugated secondary antibody to mouse immunoglobulins (Amersham International, UK). Bands were visualised as described above. An equivalent amount of total protein was loaded on to a separate mini gel consisting only of SDS–PAGE stacking gel. After electrophoresing for 30 min, depending on the contrast of the bands, gels were either scanned fresh or bands were scanned after drying the gel using a gel dryer.

To identify release of cytochrome *c* to the cytosol from the mitochondria, breast cancer cells were treated with ZOL or vehicle for 3 days and harvested on specified days by scraping. After washing the cells in ice cold PBS, they were resuspended in 500 μl of mitochondrial buffer (250 mM mannitol, 5 mM potassium dihydrogen orthophosphate, 10 mM ethylenediaminetetraacetic acid (EDTA), 5 mM 3-(N-Morpholino) propanesulphonic acid (MOPS)). Cell cytosol fraction was separated from the mitochondrial fraction by centrifugation at 13 000 r.p.m. for 15 min at 4°C and, after resuspending in the same supernatant, centrifuged again at 13 000 r.p.m. for a further 15 min as previously described (Slee *et al*, 2000 ). The soluble (cytosol) fractions were concentrated using Micron 10 microconcentrators. Aliquots of cytosol fractions with equivalent protein content (15 μg) from each sample were electrophoresed on 15% SDS–PAGE mini gels. Mouse monoclonal primary antibodies generated against cytochrome *c* (BD PharMingen, Becton Dickinson, NJ, USA) or β actin was used to immunoprobe membranes. Proteins were detected using a horseradish peroxidase-conjugated secondary antibody to mouse immunoglobulins. Bands were visualised as described above.

### DNA fragmentation assay

Breast cancer cells were incubated with [^3^H-methyl]-thymidine (0.1 μCi ml^−1^) for 9–16 h to label DNA and then washed before exposure to the indicated treatment. Cells were lysed with lysis buffer and fragmented double stranded DNA was separated from chromosome-length, unfragmented DNA followed by trichloroacetic acid (TCA) precipitation (Duke and Cohen, 1992).

### Stable transfections

MDA-MB-231 cells were transfected with pUSEamp(+) bcl-2 plasmid or a control plasmid without insert, using SuperFectTM transfection reagent (Qiagen Ltd. West Sussex, UK) according to the manufacturers' instructions. Selection of transfected clones was done using culture medium containing 2 mg ml^−1^ G418 sulphate. Expression of bcl-2 was assessed by Western blotting on whole cell lysates of selected clones using an antibody recognising murine bcl-2 (DAKO, High Wycombe, UK)

### Statistics

All experiments were performed at least twice and results shown are mean of replicate samples (*n*=4) from a single experiment. Statistical analysis of the data was carried out using analysis of variance with ANOVA: Schiffe test using Statview 4.0 software package for the Apple Macintosh. Statistical significance is indicated as **P<0.0001 compared to control unless otherwise stated.

## RESULTS

### Suppression of ZOL-induced apoptosis by bcl-2

**Figure 1:**
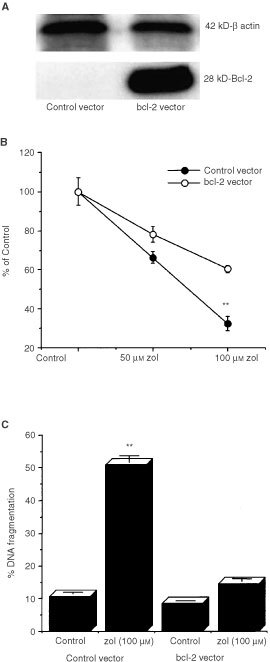
(**A**) Immunoblotting of bcl-2 protein in whole cell extracts of MDA-MB-231 clone-1 transfected with pUSEamp (+) plasmid containing wild-type mouse bcl-2 and a clone transfected with control vector. Levels of bcl-2 expression in transfected and control clones were compared relative to β actin protein. (**B**) Overexpression of bcl-2 suppresses ZOL effects on breast cancer cells. Both control vector and bcl-2 overexpressing MDA-MB-231 cells (clone 1) were incubated with 0, 50 and 100 μM ZOL for 3 days and cell viability was quantitated by MTS dye reduction assay. Clone 1 was shown to be significantly less sensitive to ZOL induced reduction in cell viability than cells transfected with control vector. Results are shown as mean±s.d. Significance was **P<0.0001 compared to bcl-2-transfected cells treated with 100 μM ZOL. (**C**) The induction of DNA fragmentation by ZOL on MDA-MB-231 cells was significantly inhibited in MDA-MB-231 clone 1, overexpressing bcl-2. Both control vector and clone 1 cells were treated with 100 μM of ZOL for 3 days. Results are shown as mean±s.d.. ZOL treatment significantly increased DNA fragmentation in control clone. **P<0.0001 (In bcl-2 transfected cells 100 μM of ZOL did not significantly increase DNA fragmentation. *P*=0.053).

To elucidate the role of bcl-2 and ZOL-induced apoptosis in breast cancer cells, we determined whether forced expression of bcl-2 was capable of abrogating ZOL-induced loss of cell viability and DNA fragmentation. Transfection of MDA-MB-231 cells with the pUSEamp (+) plasmid containing a wild-type mouse bcl-2 resulted in generation of at least two clones which stably over-expressed bcl-2. Clone 1, which had the highest expression, and a control transfected clone (C1) (Figure 1A

), were used in subsequent experiments to assess the role of bcl-2 in ZOL-induced apoptosis. The inhibitory effects of ZOL on MDA-MB-231 cell viability was suppressed by bcl-2 as clone 1 was shown to be significantly less sensitive than cells transfected with control vector (Figure 1B) or parental cells (data not shown). Following treatment with 100 μM ZOL for 3 days, loss of cell viability was significantly attenuated in cells over-expressing bcl-2 compared to control-vector transfected cells. Furthermore, in cells transfected with control-vector, 100 μM ZOL induced a five-fold increase in DNA fragmentation after 3 days of treatment compared to vehicle treated cultures (Figure 1C). However in cultures over-expressing bcl-2 this increase was less than two-fold compared to respective control.

### Cytochrome *c* release

**Figure 2:**
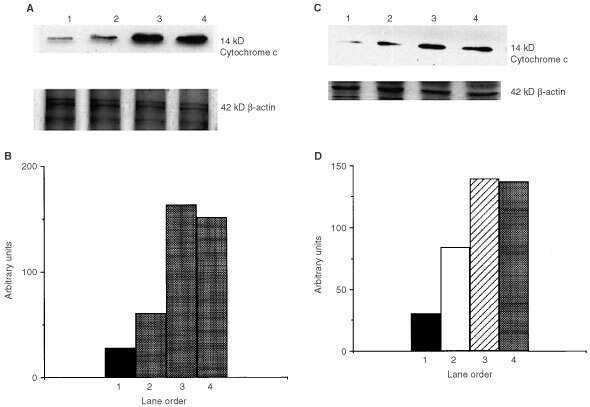
(**A**) MDA-MB-231 cells were incubated in the presence of 100 μM ZOL and cell extracts were prepared on specified days. Extracts were twice centrifuged at 13 000 r.p.m. for 15 min and the post-mitochondrial supernatant (cytosol) was concentrated as described in Materials and Methods. Cytochrome *c* levels in samples having equivalent protein contents (15 μg) were determined by Western analysis. Lane order: 1, control cultures day 3; 2, ZOL treated, day 1; 3, ZOL treated, day 2; and 4, ZOL treated, day 3. (**B**) Corresponding densitometric analysis of cytochrome *c* levels in cytosol for each day of treatment. (**C**) MCF-7 cells were incubated in the presence of 10, 50 and 100 μM ZOL and cell extracts were prepared after 3 days of above treatments. Cytosol fractions were prepared as described above and the cytochrome *c* levels in samples having equivalent protein contents (20 μg) were determined using Western analysis. Lane order: 1, control cultures; 2, 10 μM ZOL; 3, 50 μM ZOL; and 4, 100 μM ZOL. (**D**) Corresponding densitometric analysis of cytochrome *c* levels in cytosol for each treatment.

The anti-apoptotic members of the bcl-2 family of proteins inhibit mitochondrial cytochrome *c* release and caspase activation. To examine this aspect of the apoptotic pathway, MDA-MB-231 cells were treated for up to 3 days with 100 μM ZOL and MCF-7 cells were treated with 10, 50 and 100 μM of ZOL for 3 days, and the appearance of cytochrome *c* in cytosol fractions was determined by Western blot analysis. Figure 2A

and C demonstrates that there is an induction of cytochrome *c* release into the cytosol with increasing time and concentration respectively of treatment with ZOL. To our knowledge, this is the first demonstration that mitochondrial cytochrome *c* release has been shown in association with N-BP mediated apoptosis in any cell line.

### ZOL treatment induces activation of caspase 3

**Figure 3:**
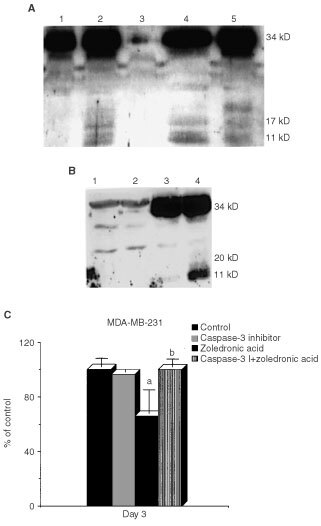
(**A**) Identification of caspase-3 activity in breast cancer cells. MDA-MB-231 cells were incubated in the presence of 100 μM ZOL for 3 days before cell extracts were prepared and Western blot analysis carried out. Two prominent cleaved products of pro-caspase-3 (indicating caspase activation) were seen at 20 and 11 kD. Lane order: 1, MDA-MB-231 cells treated with vehicle alone; 2, MDA-MB-231 treated with taxotere 10 nM for 2 days as a positive control; 3, MCF-7 treated with TNF-α 20 ng ml^−1^ for 2 days, as a negative control; 4, MDA-MB-231 treated with pamidronate 100 μM and 5, MDA-MB-231 treated with ZOL 100 μM. (**B**) Identification of caspase-3 activation by ZOL in MCF-7 cells with forced expression of caspase-3. Both control vector and caspase-3 expressing MCF-7 cells were incubated with 100 μM ZOL for 3 days before cell extracts were prepared and Western blot analysis carried out. Lane order: 1, Control vector MCF-7 treated with vehicle alone; 2, Control vector MCF-7 treated with ZOL; 3, Caspase-3 expressing MCF-7 treated with vehicle alone and 4, caspase-3 expressing MCF-7 treated with ZOL. (**C**) Attenuation of ZOL effects by a caspase-3-selective inhibitor. MDA-MB-231 cells were treated with the caspase-3 inhibitor (0.5 μM) 3 h prior to addition of 35 μM of ZOL for 24 h. Medium was then replaced with medium containing either the caspase-3 inhibitor or vehicle (DMSO) alone. On day 3 cell viability was quantitated using MTS dye reduction assay. Results are shown as mean±s.d. Significance levels were ^a^ *P*<0.0005 *vs* untreated cells, ^b^ *P*<0.0005 *vs* ZOL treated cells.

On activation pro-caspase-3 (34 kD) is cleaved into two fragments of 20 and 11 kD. To investigate the relationship of ZOL induced cytochrome *c* release on effects on caspase-3 activation, MDA-MB-231 cells were incubated for 3 days with ZOL (100 μM), cell lysates were prepared and immunoblotting was carried out with an antibody recognising pro-caspase-3 and its cleavage products. Cleavage consistent with caspase-3 activation was observed in ZOL treated cells, but only the 34 kD full-length pro-caspase-3 species was detected in control (vehicle treated) cultures (Figure 3A

). Both pro-caspase-3 and its cleaved products were absent from extracts of MCF-7 cells either untreated (not shown) or treated with TNF-α 12 ng ml^−1^ for 24 h (Figure 3A). MCF-7 cells do not express caspase-3, but are still induced to undergo apoptosis by a number of agents (Kurokawa *et al*, 1999) suggesting that cell death could be associated with activation of another, as yet unidentified caspase in these cells. To determine whether N-BPs can modulate caspase-3 in these cells, we assessed effects of ZOL on a transfected clone of MCF-7 cells stably expressing caspase-3. These cells also underwent apoptosis with ZOL treatment as assessed by loss of cell viability (data not shown) and cleavage of pro-caspase-3 consistent with activation was observed after treatment with ZOL for 3 days (Figure 3B).

To further confirm the role of caspase-3 activation in ZOL-mediated apoptosis, MDA-MB-231 cells were preincubated with a caspase-3-selective inhibitor (0.5 μM) 3 h prior to addition of ZOL (35 μM). After 24 h incubation, medium was removed and replenished with fresh medium containing only the caspase-3 inhibitor or DMSO vehicle. Results showed that the caspase-3-selective inhibitor provided full protection against cell death induced by this N-BP (Figure 3C), thus implicating activation of caspase-3 in its mechanism of action.

### Effects on membrane localisation of Ras

The initiating signal for N-BP mediated apoptosis in breast cancer cells is presently unknown. However, it has been suggested previously that N-BP-induced apoptosis in other cell types may be associated with impaired prenylation of key cellular proteins. Prenylation of Ras is required for its normal membrane localisation and function (Rowinsky *et al*, 1999).

**Figure 4:**
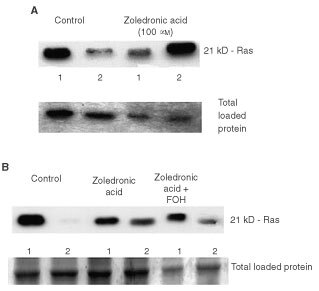
(**A**) Identification of ZOL induced reduction in active Ras (membrane bound) in MDA-MB-231 cells. MDA-MB-231 cells were treated with 100 μM ZOL for 3 days. Level of expression of Ras protein in cytosol and membrane fractions was determined by Western analysis and equivalence of loading was determined by total protein staining as described in Materials and Methods. Lane order: 1, membrane fraction; 2, cytosol fraction. (**B**) MCF-7 cells were treated with 40 μM of FOH (mixed isomers) 3 h before addition of 50 μM ZOL for 24 h. Medium was then replaced with fresh medium containing 40 μM FOH (mixed isomers) or vehicle only for a further 2 days. Level of expression of Ras protein in cytosol and membrane fractions following each treatment was determined by Western analysis as described above. Equivalence of loading was determined by total protein staining as described in Materials and Methods. Lane order: 1, membrane fraction; 2, cytosol fraction.

In order to determine if ZOL treatment affects Ras membrane localisation, MDA-MB-231 cells, which have mutated *K-ras* (Gilhooly and Rose, 1999), were incubated for 3 days with 100 μM ZOL and Ras protein levels in cytosol and membrane fractions were determined by immunoblotting. Figure 4A

demonstrates that in control cultures Ras is predominantly associated with the cell membrane fraction. However, in cultures treated with ZOL for 3 days, Ras is predominantly found in the cytosol fraction and the amount of Ras protein associated with the cell membrane is greatly reduced. These results lend further support to the suggestion that treatment of MDA-MB-231 cells with ZOL inhibits generation of FPP leading to decreased prenylation of Ras. Similar failure of Ras membrane localisation was seen with MCF-7 cells (wild-type *ras*) treated with ZOL (Figure 4B) while co-treatment with mixed isomers of farnesol (FOH) attenuated this effect.

### Farnesol (mixed isomers) provides protection against ZOL-induced loss of cell viability

**Figure 5:**
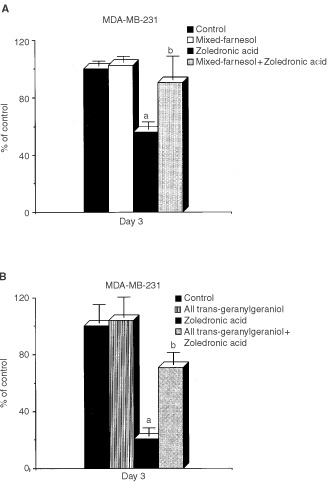
(**A**) Effects of FOH (mixed isomers) on ZOL-induced inhibition of cell viability. MDA-MB-231 cells were treated with or without 40 μM of FOH (mixed isomers) 3 h before addition of 40 μM ZOL for 24 h. Medium was then replaced with fresh medium containing 40 μM FOH (mixed isomers) or vehicle (ethanol) only for a further 3 days. Results are shown as mean±s.d. Significance ^a^ *P*<0.0001 *vs* vehicle treated cells (control). ^b^ *P*<0.0001 *vs* ZOL alone treated cells. Control *vs* ZOL + FOH was not significant (*P*=0.86). (**B**) Effects of all *trans-*GGOH (40 μM) on ZOL induced inhibition of cell viability. MDA-MB-231 cells were treated with or without 40 μM all *trans*-GGOH for 3 h before exposing to 40 μM of ZOL for 24 h. Medium was then replaced with fresh medium containing 40 μM all *trans*-GGOH alone or vehicle only. Results are shown as mean±s.d. Significance ^a^ *P*<0.0001 *vs* vehicle treated cells (control). ^b^ *P*<0.0014 *vs* ZOL alone treated cells. Viability of cultures treated with ZOL+GGOH was significantly lower than control (*P*<0.01).

In order to assess the efficacy of various isoprenoids to attenuate ZOL-mediated apoptosis, we determined effects of co-treatment with farnesol (FOH mixed isomers) and all *trans* geranylgeraniol (GGOH) on ZOL-induced loss of cell viability. MDA-MB-231 cells were treated with 40 μM of each isoprenoid for 3 h prior to addition of ZOL. After 24 h co-treatment, media were removed and replenished with fresh media containing isoprenoid or ethanol vehicle only for a further 2 days. At the end of the treatment period, cell viability was assessed by MTS assay. Co-treatment with FOH (mixed isomers) protected against ZOL-induced apoptosis, restoring viability to approximately 90% of control cultures. Co-treatment with GGOH provided partial protection, since viability of cultures co-treated with ZOL+GGOH was approximately 70% of respective controls (Figure 5A,B

). This study suggests that impaired protein farnesylation may play a more important role in N-BP mediated apoptosis in breast cancer cells than failure of protein geranylgeranylation.

## DISCUSSION

There is increasing evidence that BPs may have direct inhibitory effects on breast cancer cells. These may be related to decreased cell adhesion since pretreatment of MDA-MB-231 breast cancer cells with BPs (ibandronate, olpandronate, pamidronate and alendronate) prevents attachment and spreading of cells onto bone slices (van der Pluijim *et al*, 1996; Boissier *et al*, 1997). Animal studies have demonstrated that pretreatment of nude mice with BPs before inoculation of tumour cells reduces the development of osteolytic lesions (Sasaki *et al*, 1995). Furthermore, a recent study using an *in vitro* model of cell invasion has suggested that BPs may inhibit this early event in the formation of bone metastases (Boissier *et al*, 2000). We have shown that BPs directly induce apoptosis in breast cancer cells and this observation has recently been confirmed by other studies (Fromigue *et al*, 2000; Hiraga *et al*, 2001; Jagdev *et al*, 2001). Our present analysis of the apoptotic pathways affected by ZOL provides insight into the mechanism by which N-BPs induce cell death in breast cancer cells. The bcl-2 family of pro- and anti-apoptotic proteins plays an important role in apoptosis induced by a large variety of stimuli (Korsmeyer, 1999). It has been suggested that bcl-2 prevents the release of cytochrome *c* into the cytosol, thereby inhibiting the activation of caspases (Marzo *et al*, 1998). An aim of the present study was to clarify the involvement of bcl-2 and cytochrome *c* release in ZOL mediated caspase activation in breast cancer cells. We have already shown that bcl-2 protein is down-regulated upon treatment of breast cancer cells with N-BPs (Senaratne *et al*, 2000) and now report that forced expression of bcl-2 in MDA-MB-231 breast cancer cells efficiently inhibits ZOL-induced DNA fragmentation, which is a consequence of caspase activation in breast cancer cells. These results are in agreement with a study carried out on myeloma cells demonstrating that forced expression of bcl-2 is capable of abrogating BP induced apoptosis (Aparicio *et al*, 1998). We now demonstrate for the first time that treatment of breast cancer cells with ZOL is associated with release of mitochondrial cytochrome *c* into the cytosol.

Support for the role of caspase activation in N-BP-induced apoptosis in breast cancer cells is provided by demonstration of PARP cleavage (Senaratne *et al*, 2000) as well as abrogation by z-VAD-fmk (a broad-spectrum caspase inhibitor) of MCF-7 cell growth inhibition induced by four BPs (zoledronic acid, ibandronate, pamidronate and clodronate, Fromigue *et al*, 2000). Activation of caspase-3-like proteases is thought to be an irreversible step in the pathway leading to DNA fragmentation in apoptotic cell death (Cohen, 1997). Hiraga *et al* (2001) have demonstrated that a selective caspase-3 inhibitor is capable of blocking ibandronate-induced DNA fragmentation in MDA-MB-231 breast cancer cells and we have similarly found that caspase-3 inhibition significantly protects against ZOL-induced loss of viability in these cells.

Furthermore, in the present study we have presented direct evidence for activation of this cell death protease by ZOL in MDA-MB-231 breast cancer cells by immunoblotting. We and others (Fromigue *et al*, 2000; Senaratne *et al*, 2000; Jagdev *et al*, 2001) have demonstrated that N-BPs are capable of inducing loss of cell viability and DNA fragmentation in MCF-7 cells which are caspase-3 null (Kurokawa *et al*, 1999). However, our present study illustrates that in MCF-7 cells stably transfected with caspase-3, ZOL is capable of activating this cell-death protease. We suggest that in parental MCF-7 cells, treatment with N-BPs is associated with activation of another caspase, possibly caspase 7 (Puig *et al*, 2001).

How does treatment of breast cancer cells with N-BPs lead to cytochrome *c* release and caspase activation? In order to address this question we evaluated the possible role of impaired protein prenylation. Recent pharmacological studies suggest that N-BPs such as pamidronate, alendronate and risedronate act on the cholesterol biosynthetic pathway (Luckman *et al*, 1998; Shipman *et al*, 1998). N-BPs such as ZOL have recently been shown to inhibit farnesyl pyrophosphate synthase (van Beek *et al*, 1999a; Dunford *et al*, 2001) thus blocking the production of the pyrophosphate intermediates FPP and GGPP. These intermediates are important for post-translational modification of key regulatory proteins. Prenylation of small G proteins such as Rac, Rho and various members of the Rab family occurs through transfer of either one or two 20 carbon geranylgeranyl isoprenoids from GGPP to these proteins (reviewed in Rowinsky, 1999) by the action of geranylgeranyl transferases (GGTases). Another key prenylated protein is Ras, which undergoes farnesylation by the addition of a 15-carbon farnesyl isoprenoid from FPP by the action of farnesyl transferase (FTase). Failure of FPP and GGPP generation by inhibition of FPP synthase would thus be expected to lead to impaired prenylation of these important regulatory proteins.

Ras proteins play pivotal roles in the control of normal and transformed cell growth. While *ras* mutations are not as common in breast cancer as in some other types of malignancy, the increased activity of growth factor signalling pathways which are mediated via Ras proteins makes targeting Ras action an attractive option in breast cancer treatment. By preventing maturation into its biologically active form, farnesyl transferase inhibitors (FTIs) abolish the membrane localisation (Lerner *et al*, 1995) and function of Ras (Nagase *et al*, 1996) and these agents are currently under evaluation in clinical trials in patients with breast cancer (Johnston and Kelland, 2001). We have determined if N-BPs can also prevent Ras processing leading to its cytoplasmic accumulation. In MDA-MB-231 and MCF-7 cells, treatment with ZOL for 3 days clearly prevented membrane localisation of Ras as determined by immunoblotting. Therefore our results suggest for the first time that N-BPs inhibit Ras processing and membrane localisation in breast cancer cells in a manner similar to FTIs.

To investigate the role of protein prenylation in N-BP mediated apoptosis in breast cancer cells further, we determined if replacement of intracellular isoprenoids could rescue breast cancer cells from ZOL-induced apoptosis. In most studies designed to investigate this potential mechanism in osteoclasts and macrophages, the ability of FOH and GGOH to prevent effects of N-BPs have been utilised. These analogues of FPP and GGPP have been reported to display greater membrane permeability and be converted to the respective pyrophosphate forms intracellularly by a salvage pathway (Crick *et al*, 1997). In osteoclasts, N-BP-induced apoptosis was attenuated by addition of GGOH but not FOH (Fisher *et al*, 1999; van Beek *et al*, 1999b). However, in J774 macrophages undergoing N-BP induced apoptosis, addition of either FOH or GGOH (Benford *et al*, 1999) inhibited caspase-3 activation and induction of apoptosis. Our present study in breast cancer cells has shown that FOH (mixed isomers) both protects MDA-MB-231 cells from ZOL-induced inhibition of cell viability and attenuates ZOL-induced loss of Ras membrane localisation in MCF-7 cells. This suggests that Ras is one important prenylated protein that is altered in activity by N-BP treatment of breast cancer cells. We found that co-treatment with GGOH was less effective than FOH in protecting MDA-MB-231 cells from ZOL-induced apoptosis. This is in contrast to a recent study by Jagdev *et al* (2001) who reported that co-treatment with GGOH rescued MCF-7 cells from ZOL induced apoptosis whereas FOH had little effect.

Taken together our present results demonstrate that ZOL induces apoptosis in human breast cancer cells. This apoptosis is associated with release of cytochrome *c* into the cytosol and subsequent activation of the caspase cascade. These effects are abrogated by forced expression of bcl-2. Our results implicate a role for protein prenylation and impaired Ras membrane localisation in apoptosis. Further research is likely to provide valuable insights into the role played by prenylated proteins in the initiation of these apoptotic pathways. Currently BPs are used in the treatment of patients who develop bone metastases from carcinoma of the breast. The primary aim here is to reduce the skeletal morbidity such as bone pain, fracture and hypercalcaemia (Hortobagyi *et al*, 1996; Body *et al*, 1998). At present, the report by Diel *et al* (1998) is the only one to demonstrate a survival advantage with the use of clodronate in the adjuvant setting. With the advent of the newer and more potent BPs such as ZOL, the potential to make an impact on progression-free and overall survival may be achieved. Knowledge of the various pathways involved may facilitate additional therapeutic interventions to maximise the effects of BPs.
