# The bisphosphonate zoledronic acid impairs membrane localisation and induces cytochrome *c* release in breast cancer cells

**DOI:** 10.1038/sj.bjc.6600571

**Published:** 2002-11-12

**Authors:** S G Senaratne, J L Mansi, K W Colston

## Abstract

*British Journal of Cancer* (2002) **87**, 1340–1340. doi:10.1038/sj.bjc.6600571 www.bjcancer.com

© 2002 Cancer Research UK

**Correction to:** *British Journal of Cancer* (2002) **86**, 1479–1486. doi:10.1038/sj.bjc.6600297

## 

In the above paper an error occurred in the title.

The correct title is printed below:

The bisphosphonate zoledronic acid impairs Ras membrane localisation and induces cytochrome *c* release in breast cancer cells

